# East meets west: using ethnobotany in ethnic urban markets of Barcelona metropolitan area (Catalonia) as a tool for biocultural exchange

**DOI:** 10.1186/s13002-023-00636-x

**Published:** 2023-12-17

**Authors:** Ugo D’Ambrosio, Cristina Pozo, Joan Vallès, Airy Gras

**Affiliations:** 1 Laboratori de Botànica, Unitat Associada CSIC, Facultat de Farmàcia i Ciències de l’Alimentació . IRBio, Universitat de Barcelona, Avinguda Joan XXIII 27-31, 08028 Barcelona, Catalonia Spain; 2 Institut Botànic de Barcelona (IBB), CSIC-Ajuntament de Barcelona, Passeig del Migdia s/n, Parc de Montjuïc, 08038 Barcelona, Catalonia Spain; 3 Global Diversity Foundation (GDF), 37 St. Margaret’s Street, Canterbury, Kent, CT1 2TU England, UK; 4 Institut d’Estudis Catalans, Carrer del Carme 47, 08001 Barcelona, Catalonia Spain

**Keywords:** Applied ethnobotany, Biocultural learning, Chinese diaspora, Chinese Pharmacopoeia, Ethnic food stores, Metropolitan area of Barcelona, Urban ethnobotany

## Abstract

**Background:**

Ethnobotanical studies in metropolitan areas and urban ethnic markets have grown considerably in recent years as large cities have demonstrated to be significantly rich in biocultural diversity and in driving its evolution, as human populations migrate from one region to another. Urban spaces also represent important places of rich multicultural and multilingual interaction and exchange, where ethnobotany can act as a bridge between research and action. The purpose of this study is to present a case study on how to use ethnobotany in multicultural urban settings by studying people-plant interactions and the larger implications and applications to promote biocultural learning in these areas.

**Methods:**

We inventoried the botanical composition of fresh and dry products sold in most food stores owned by Chinese immigrants in Fondo, a neighbourhood of Barcelona’s metropolitan area, in Santa Coloma de Gramenet municipality (Barcelonès county, Catalonia, Iberian Peninsula), pharmacologically validating the obtained list with the Chinese Pharmacopoeia. We also participated in multiple dissemination activities and materials (non-academic and academic), along with exchanges with the broader community in relation to this research.

**Results:**

In total, 103 plants were identified at the species level, pertaining to 88 genera and 46 botanical families. Including the infraspecific level, a total of 113 plant taxa were inventoried. One algal and six fungal species were also recorded, but not included in the analyses. Brassicaceae (12.4%) and Fabaceae (10.6%) were the most predominant families inventoried, followed by Cucurbitaceae (7.1%) and Poaceae (7.1%). Over three-quarters of all the taxa have an Asian origin (76.11%), indicating a high conservation of the use of Asian taxa. Over one-third (36.89%) of the plant parts pertain to species contained in the Chinese Pharmacopoeia, showing the relevance of medicinal plants in local stores and the preponderance of Eastern Asian food-medicine continuums. To promote ethnobotanical education programmes, over 50 dissemination activities and educational materials were produced from this study and shared with the local urban community in different fora.

**Conclusions:**

Further research in these and similar settings can provide significant ethnographic information to better understand anthropological processes and phenomena underlying migration and transculturation that can be used in an umbrella of applications, from adequate nomenclature and labelling of foreign products in local languages to multicultural integration and social cohesion programmes along with educational activities on biocultural topics.

**Supplementary Information:**

The online version contains supplementary material available at 10.1186/s13002-023-00636-x.

## Background

The metropolitan area of Barcelona has seen a considerable increase in foreign populations in the past three decades, shifting from less than 3% of the total population in the early 2000s, to around 16% in 2022, with a predominance of immigrants from Morocco, Italy, and China, the latter being the focus of this study. In total, more than 170 nationalities inhabit the metropolitan area [1], figures which are probably underestimates. The rapid raise of immigrant populations in the study area, from less than 5% in the 1990’s to representing over a fifth of the population in the 2020’s [1], has been accompanied with a rise in ethnic food stores and new plant products not seen decades ago due to the lack of large numbers of foreign communities. Such abundance and relative novelty, linked to the limited intercultural bridges between migrant populations and the negotiations amongst them, make these urban environments ethnobotanically and ethnographically rich, worth investigating further. This has been extensively shown with migrants’ medicinal floras [2, and references therein], yet much less with food plants.

As ethnobiology continues to evolve as a discipline, studies in the last two decades have increasingly expanded to urban areas, showing that biocultural diversity is not restricted to remote areas on the planet but is very much part of metropolises, their inhabitants and their evolutions [3–5]. In parallel, related studies provide a surprising amount of ethnobiological and particularly ethnopharmaceutical information from human migrations and immigrant communities, in multiple settings worth considering for their richness and relevance in biocultural terms (e.g., [2, 6–8]).

In addition to the ethnobiology of cities, migration and intercultural exchanges and evolution, much has also been written on the food-medicine continuum [9] and the concept of folk functional foods and nutraceuticals [10], especially in ethnobotanical and cognate literature and foci. The food-medicine continuum is widespread amongst most cultural traditions and of profound importance in traditional Chinese medicine (TCM) where dietary therapy and herbal medicine are central to balanced health and wellbeing [11, 12], with clear boundaries often difficult to distinguish. Following the School of Naturalists (*Yinyangjia*), which combined the five elements theory with the yin–yang theory, different diets and medicines are associated with distinct qualities and meridians in this ancient ethnomedicinal system [13, 14], still highly observed by people with Chinese descent.

Ethnobotany has also been deemed as a valuable social and environmental educational tool [15, 16] as well as for promoting biocultural sustainability in the youngest generations [17]. Though ethnobotany as an educational tool has been used more profusely in rural areas and with the youth, the benefits of employing it in urban and peri-urban ones or with elders have been dismissed. Therefore, ethnobotany can play a significant role in mobilizing knowledge related to biocultural systems, along with learning and change, and its value in building sustainability and resilience of local food systems and identities should not be overlooked.

Based on these premises regarding the ethnobotany and ethnopharmacology of migrant populations in urban areas, and the biocultural implications and applications that these human-plant movements entail, the aim of this research is to study the richness of plants sold in ethnic Chinese stores in the conurbation of Barcelona, and how this information has been used to propose and design socioenvironmental education and integration programmes in a multicultural urban setting as well as to produce multiple academic and non-academic outputs relating to this research. To do so, three main specific objectives are considered: (1) to inventory and describe the plants (culinary and medicinal) of a series of ethnic Chinese stores in Fondo neighbourhood, (2) to pharmacologically validate the obtained list of plants using the 2015 edition of the Chinese Pharmacopoeia, and (3) to explain past, present and potential future applications (and implications) of this study for urban ethnobotanical education, civil participation and multicultural integration in Santa Coloma de Gramenet municipality.

## Methods

### Study and intervention site

**Fig. 1:**
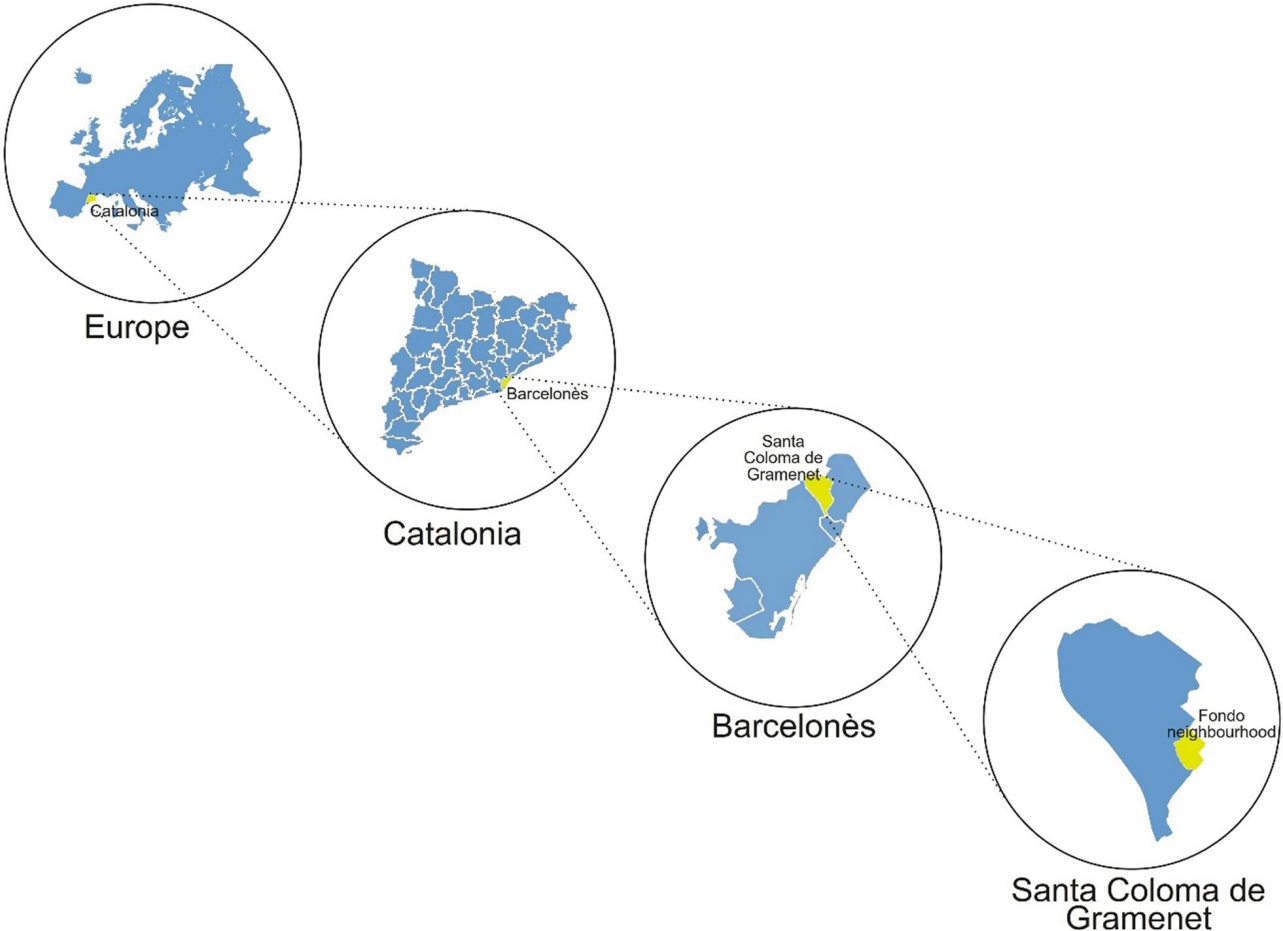
Map of the study area

The botanical inventory and educational programme were carried out in the greater metropolitan area of Barcelona (Catalonia, Iberian Peninsula), concretely in Fondo neighbourhood of Santa Coloma de Gramenet municipality (Fig. 1).

With a population that has remained quite constant over the past 2–3 decades around 118,000 inhabitants, Santa Coloma de Gramenet has seen a continued rise of foreign population, from representing less than 5% of the total population in the 1990s to representing over a fifth (21.59%) of the population in 2022 [1]. With 83 nationalities, Santa Coloma de Gramenet is one of Barcelona’s conurbation municipalities with more foreign population of Catalonia [1]. While the percentage of the foreign population of Chinese origin in Catalonia is relatively low (4.97%), it is much higher in Santa Coloma de Gramenet (18.99%) and especially in the Fondo neighbourhood (41.57%), representing one of the conurbations in Catalonia with a higher proportion of inhabitants born in China (Table 1).

**Table 1:** Total population, total foreign population and that of Chinese origin in Catalonia, Barcelonès county, Santa Coloma de Gramenet municipality, and Fondo neighbourhood in 2022

Unit	Catalonia	Barcelonès county	Santa Coloma de Gramenet municipality	Fondo neighbourhood
Total population	7,770,689	2,280,042	117,981	16,561
% of population with respect to the total	*100*	*29.34*	*1.52*	*0.21*
Foreign population	1,271,810	483,968	25,470	7203
% of foreign population with respect to the total	*16.37*	*21.23*	*21.59*	*44.49*
Foreign population of Chinese origin	63,228	34,375	4,837	2994
% of foreign population of Chinese origin	*4.97*	*7.10*	*18.99*	*41.57*
% of Chinese origin with respect to the total	*0.81*	*1.51*	*4.10*	*18.08*

Data for year 2022, taken from Statistical Institute of Catalonia [1]

Italics correspond to percentual values

Most Chinese immigrants in the metropolitan area of Barcelona come from the Zhejiang province, chiefly from the south-eastern Qingtian county and are speakers of Wu Chinese [18]. Most Chinese immigrants in the metropolitan area of Barcelona speak to varying degrees Catalan and Spanish, with youngest generations, with scholarship in the area, being more fluent.

### Data collection and analysis

The results presented in this research were initiated in 2015 with the final bachelor’s degree project of one of the co-authors, for the Faculty of Pharmacy and Food Sciences, University of Barcelona [19], and expanded until today with a series of activities and materials based on this original project, the stores inventoried, and the institutional collaborations arising from it.

**Fig. 2:**
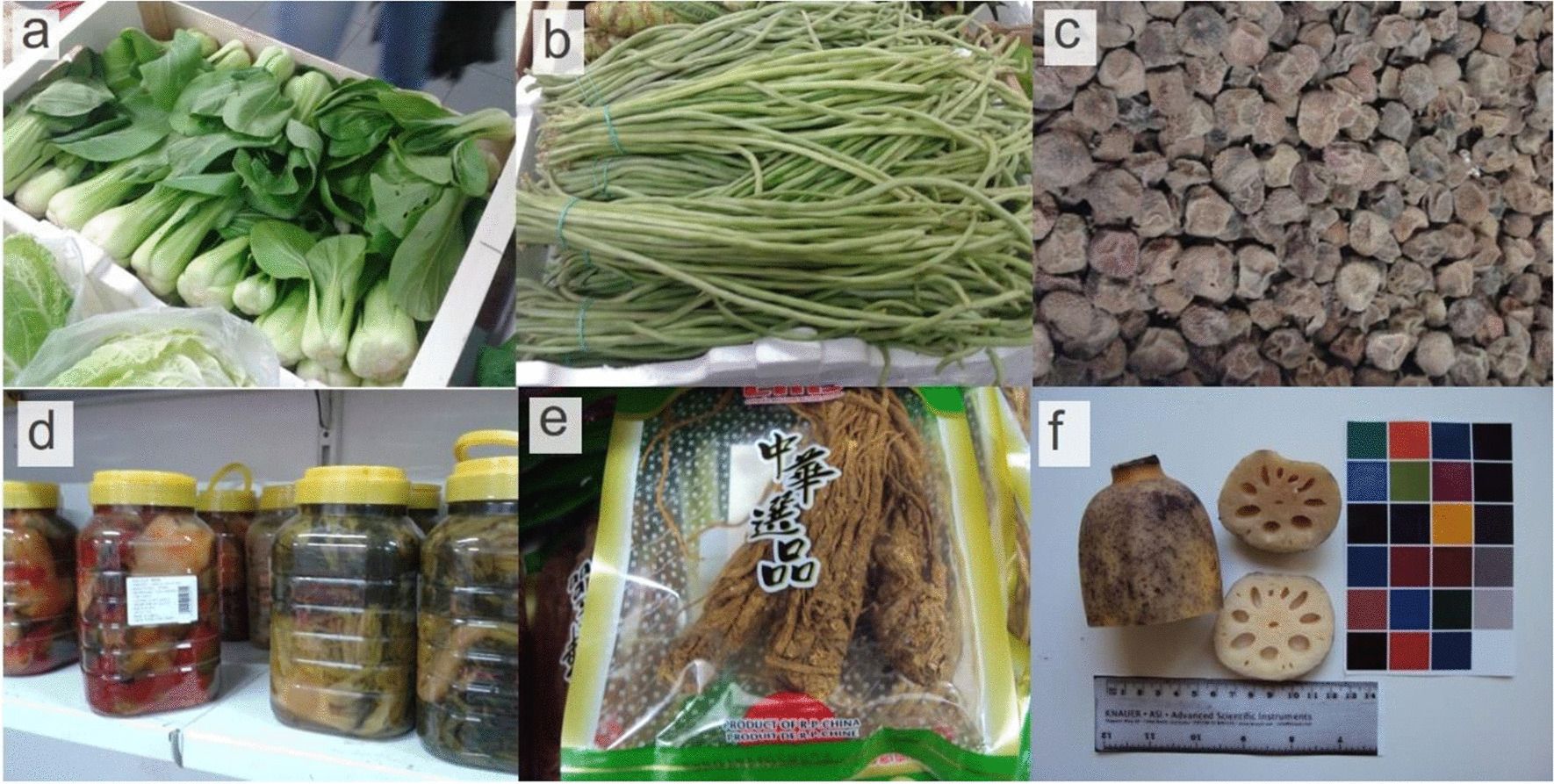
Photographies of a selection of products. **a** Fresh pak choi (*Brassica rapa* subsp*. chinensis)*; **b** Fresh asparagus bean (*Vigna unguicularis* subsp. *sesquipedalis*); **c** Dried litchi (*Litchi chinensis*); **d** Preserved *Brassica* spp.; **e** Packaged female ginseng root (*Angelica sinensis*); **f** Lotus rhizome (*Nelumbo nucifera*)

Five stores owned and frequented by Chinese expatriates were randomly selected and inventoried in this study including more than half of the food stores in the neighbourhood of Fondo owned by Chinese immigrants. We inventoried plants during 15 visits to the stores in different seasons of the year from 2015 to 2019, and three additional visits were carried out in 2020, to collect remaining voucher specimens as well as some supplementary information. All kinds of plants were considered, either fresh, dry or otherwise transformed (fermented, processed), although preference was given to fresh and dry products. Food and medicinal plants were collected equally, with plants also serving both functions. We also carried out informal conversations with sellers and buyers, although we did not do any ethnographic data collection at this stage of the project. Images of the data collection phase are given in Fig. 2.

Once the botanical samples were brought back to the Botany Laboratory (University of Barcelona), non-succulent plant parts were pressed and succulent parts were dried in a cultivation oven at 55–60 °C for 12 to 36 h depending on the amount of water present in the sample. Voucher specimens and samples were stored in Herbarium BCN (Centre de Documentació de Biodiversitat Vegetal, Universitat de Barcelona) and identified by the authors. Species and subspecies follow the nomenclature provided by multiple sources depending on if they exist in the Catalan local flora [20–24] or not [25–27], while botanical families follow APG IV [28]. Even though the research was not ethnography-based, since no ethnobotanical interviews were performed, but just punctual questions in some cases, it followed the basic principles of the International Society of Ethnobiology Code of Ethics [29], obtaining free and prior informed consent when necessary and assuring anonymity and confidentiality. In addition to sampling, a species and products photographic archive was produced (Fig. 2a–f).

After the plants were inventoried, sampled, identified and stored, we analysed their ethnobotanical characteristics including taxonomy (botanical families, genera, species and infraspecific taxa), life form (herb, liana, shrub or tree), level of preparation (fresh, dried or preserved), plant part/s sold (aerial parts, leaves, stems, roots, rhizomes, bulbs, tubers, flowers, floral stems, fruits, seeds, barks), and centre of domestication (for crops, taken from [30]) or diversification (for wild species, taken from [31]). Subsequently, we performed a bibliographic pharmacological validation against the 1995 [32] and 2015 [12] editions of the Chinese Pharmacopoeia. These calculations responded to our first and second objectives of this study.

### Academic and non-academic collaborations

The municipality of Santa Coloma de Gramenet has grown, during recent years, into a University City with a campus fully dedicated to the study of nutrition, gastronomy and food sciences, the Torribera Food Studies Campus of the University of Barcelona. In parallel, a new public library was inaugurated in late 2014 in Fondo neighbourhood, specializing in food and gastronomy with over 2000 books on these matters and a large kitchen and exhibition space for demonstrations, lectures and other social interactions. The educational and outreach component of this research, covered by objective 3 of this research, was possible thanks to a series of participants and project partners, detailed in Additional file 1.

This institutional ecosystem sets up a new environment to study the interactions between humans and food plants with a diversity of stakeholders. Amongst the various activities promoted by these organisations at the local level, two are distinctively linked to food and people: the *Flavours of the world* activities, festival and materials, in which our research group has collaborated for two consecutive years, and the *Cuisines of the world* space at the local library, conceived as a gastro-literary lab [33], where one dissemination activity was carried out by our team. The current study benefits from these initiatives and complements them by bringing a novel ethnobotanical component with a series of academic and non-academic contributions on the fringes of gastronomy, plants, migration and cities.

## Results and discussion

### Ethnobotanical characteristics of the inventoried plants

A total of 103 plants were identified at the species level, pertaining to 88 genera and 46 botanical families. Including the infraspecific level, 113 edible and/or medicinal plant taxa were recorded (Additional file 2). One algal and six fungal species were also inventoried and sampled, but not included in the analysis of this manuscript.

**Fig. 3:**
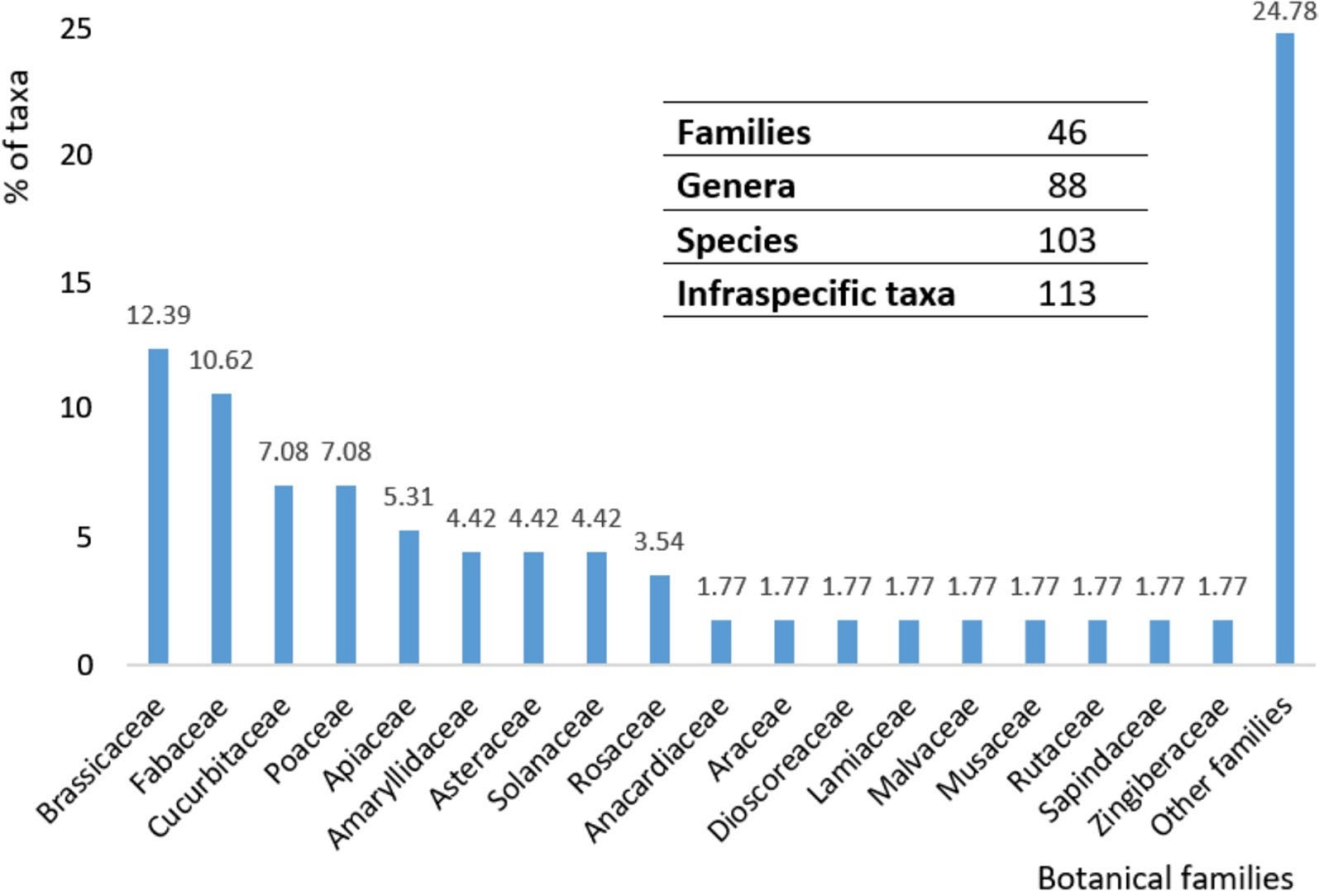
Inventoried families according to number of specific and infraspecific taxa present in the stores prospected, ordered in decreasing order. Taxa with more than 1% of the inventoried plants belonged to 18 families, while those with less belonged to 28 other families

Out of a total of 46 families, 18 hosted more than 1% of the inventoried taxa, and 28 other included less than 1%. Many of the taxa found are either not at all common or unknown in the Mediterranean, with an abundance of Brassicaceae (12.39%) and Fabaceae (10.62%), followed by Cucurbitaceae (7.08%), Poaceae (7.08%) and Apiaceae (5.31%) (Fig. 3).

According to the life form of the inventoried taxa, almost two-thirds of the plants correspond to herbs (63.72%, including one palm and one cactus), followed by lianas (15.93%), trees (13.27%) and shrubs (7.08%). The abundance of lianas is rather relevant if compared to other culinary and medicinal traditions, where they tend to be amongst the least used life forms [34, 35]. While most plants are sold exclusively fresh (49.56% of taxa) or dry (38.94%), a combination of levels of preparation was also observed (8.85%, mainly fresh and dry), followed by plants exclusively preserved (2.65%) (Additional file 2). Most stores have fresh products closer to the entrance while dry products and explicitly-considered medicines are found further back.

Only one plant part is sold in the majority of taxa (93.81%) while for a minority two plant parts are sold separately (6.19%), sometimes with different levels of preparation. A predominance of fruits (29.17%) and seeds (15.83%) is observed, followed by aerial parts and roots with 13.33% of taxa each. Leaves, rhizomes, flowers and inflorescences, bulbs, stems, and tubers followed, with percentages ranging from 9.17% to 1.67%, and barks, floral stems and seedlings represented less than 1% of the studied sample.

The inventoried ethnoflora indicates a high presence of Asian taxa, with more than one-third of the plants with their centres of origin and diversification in China (36.28%) and over three-quarters of all the taxa with an Asian origin (76.11%) [21, 22] (Additional file 2). American plants follow, with over 12% of the inventoried plants, while other plants with an exclusively African and Mediterranean/Middle Eastern origin only represent, altogether, a bit less than 12% of taxa. Therefore, a great number of plants were exotic, with no close representatives in the local Catalan flora [20–23].

This clearly supports the notion that ethnic markets play a key role in cultural practices’ production and reproduction, as has been documented in other urban areas of the world [2]. Chinese communities highly conserve such praxes, importantly linked to their local plants [36], as do other migrant communities. Similar research has directed the attention to the role of ethnic markets in cultural sustainability [37], the consumption of home foods and cooking for emotional stability and reassurance [38] and where foods overcome cultural boundaries, their meanings are reshaped and culinary hybridization and innovation happen [39]. Although not included in this research, another phenomenon of interest qualitatively observed during the prospection is the exchange between Asian, African and American immigrants in the diversity of ethnic stores specialized in common food of at least one of the three continents.

From the inventoried plants, various groups stood out, especially for their culinary uses, but also for their medicinal and nutritional properties, including fresh green leaves, leguminous seeds, condiments and spices, alliums and marshy plants. Fresh green leaves, such as the brassicas (*Brassica* spp.) and other green leaves (*Glebionis* spp., *Spinacia oleracea* L.), which represent almost 10% of the inventoried ethnoflora, are profuse in stores all year round and are usually cooked either by boiling or by stir-frying. Edible leguminous seeds and fruits also stand out, representing almost 9% of the taxa, and alliums (e.g., *Allium ampeloprasum* L. var. *porrum* (L.) J.Gay, *A. cepa* L., *A. sativum* L., *A. schoenoprasum* L. and *A. tuberosum* Rottler ex Spreng.) and marshy plants (e.g., *Nasturtium officinale* R.Br., *Eleocharis dulcis* (Burm.f.) Trin. ex Hensch., *Trapa natans* L., *Nelumbo nucifera* Gaertn. and *Euryale ferox* Salisb.), representing almost 5% of the taxa each. A significant number of condiments and spices were also reported, especially in dry form and used in teas or to season foods. In addition, plants exclusively used for their medicinal properties constituted another group of significant plants in the visited stores, also in most cases dried.

When compared to the limited research in other parts of the world, the present dataset on food plants sold in Chinese shops, points out to a conservatism of taxa used. For instance, if we compare our dataset with the plants used by Chinese and Taiwanese immigrants in Atlanta (USA), we find that two-thirds of the plants inventoried in their study were also found in Fondo’s stores [36]. While an abundance of literature exists on the plant Pharmacopoeias of migrant populations [2, and references therein], including urban and suburban areas, little has been researched in culinary plants, either of Chinese origin or not. Nonetheless, several of the processes of adaptation, maintenance, replacement and hybridization seen in medicinal uses [40, 41] could shade light on similar processes occurring in food plants.

### Ethnopharmacological validation with the Chinese Pharmacopoeia

When performing the ethnopharmacological validation of the plant parts identified at Fondo stores, over one-third of the inventoried species (36.89%, i.e., 38 species) are included in the Chinese Pharmacopoeia [12, 32] (Table 2), indicating the significance of these plants for Chinese ex-pats even in stores mostly dedicated to the sale of food. The absence of TCM-specialized stores in Barcelona’s metropolitan area might also explain this phenomenon.

**Table 2:** Inventoried species pharmacologically validated in the Chinese Pharmacopoeia (ChP) [12, 32]

Species	Pinyin transliteration^a^	Chinese Pharmacopoeia (1995*–2015)
Inventoried species and parts coinciding with ChP (included in the calculations of this paper)		
*Allium sativum*	Dausan	*Allii Sativi Bulbus*
*Angelica sinensis*	Danggui	*Radix Angelicae Sinensis*
*Astragalus propinquus* (under *A. membranaceus*)	Huangqi	*Radix Astragali*
*Atractylodes macrocephala*	Baizhu	*Rhizoma Atractylodis Macrocephalae*
*Benincasa hispida* var. *chieh-gua*	Dongguapi	*Exocarpium Benincasae*
*Capsicum annuum*	Lajiao	Fructus Capsici*
*Cinnamomum cassia*	Guizhi	*Cortex Cinnamomi*
*Codonopsis pilosula*	Dangshen	*Radix Codonopsis*
*Coix lachryma-jobi*	Yiyiren	*Semen Coicis*
*Crataegus pinnatifida*	Shanzha	*Fructus Crataegi*
*Dimocarpus longan*	Longyanrou	*Arillus Longan*
*Dioscorea oppositifolia* (under *D. opposita*)	Shanyao	*Rhizoma Dioscoreae*
*Euryale ferox*	Qianshi	*Semen Euryales*
*Glycine max*	Dandouchi	*Semen Sojae Preparatum*
*Glycyrrhiza uralensis*	Gancao	*Radix Glycyrrhizae*
*Illicium verum*	Bajiao Huixiangyou	*Fructus Anisi Stellati*
*Isatis tinctoria* (under *I. indigotica*)	Qingdai (Daqingye), Banlangen	*Radix Isatidis*
*Juglans regia*	Hetaoren	*Semen Juglandis*
*Lablab purpureus*	Baibiandou	Lablab Semen Album*
*Ligusticum striatum* (under *L. chuanxiong*)	Chuanxiong	*Rhizoma Chuanxiong*
*Lilium brownii*	Baihe	*Bulbus Lilii*
*Litchi chinensis*	Lizhihe	*Semen Litchi*
*Lonicera japonica*	Jinyinhua	*Caulis Lonicerae*
*Nelumbo nucifera*	Lianzixin/Lianzi/Lianfang/Lianxu/Oujie	*Plumula Nelumbinis, Semen Nelumbinis, Receptaculum Nelumbinis, Stamen Nelumbinis, Rhizomatis Nodus*
*Paeonia lactiflora*	Chishao	*Radix Paeoniae Alba*
*Panax ginseng*	Renshen	*Radix Ginseng*
*Prunella vulgaris*	Xiakucao	*Spica Prunellae*
*Prunus persica*	Taoren	*Semen Persicae*
*Raphanus sativus*	Laifuzi	*Semen Raphani*
*Rehmannia glutinosa*	Dihuang	*Radix Rehmanniae*
*Rosa rugosa*	Meiguihua	*Flos Rosae Rugosae*
*Salvia miltiorrhiza*	Danshen	*Radix Salviae Miltiorrhizae*
*Scaphium affine (*under *Sterculia lychnophora*)	Pangdahai	Semen Sterculiae Lychnophorae*
*Sesamum idicum*	Heizhima	*Oleum Sesami*
* Siraitia grosvenorii* (under *Momordica grosvenorii*)	Luohanguo	*Fructus Siraitiae*
*Vigna angularis* (under *Phaseolus angularis*)	Chixiaodou	*Semen Vignae*
*Zingiber officinale*	Ganjiang	*Rhizoma (Recens) Zingiberis*
*Ziziphus jujuba*	Dazao	*Fructus Jujubae*
Inventoried species (but not their parts) coinciding with ChP (not included in the calculations of this paper)		
*Allium tuberosum*	Jiucaizi	*Semen Allii Tuberosi*
*Brassica juncea*	Jiezi	*Semen Sinapis*
*Daucus carota*	Nanheshi	*Fructus Carotae*
*Hordeum vulgare*	Maiya	*Fructus Hordei Germinatus*
*Lycium chinense*	Digupi	*Cortex Licii*
*Oryza sativa*	Daoya	*Fructus Oryzae Germinatus*

All the non-pharmacopoeia medicines do not appear in the table. Drug names are given in pharmaceutical Latin

*Pharmaceutical drugs included in Chinese Pharmacopoeia 1995 [32]

^a^For standard Mandarin names, Roman transliteration, Hanyu pinyin without tonal (diacritical) marks, is used

Of the 38 species and plant parts coinciding with the Chinese Pharmacopoeia’s plant-based pharmaceutical drugs, a high diversity of families was observed (a total amount of 27), with Fabaceae being the most relevant (five species, 13.51% of coincidences). Rosaceae followed with 8.11% (three species) and Apiaceae, Cucurbitaceae, Lamiaceae and Sapindaceae with 5.41% each (two species). The remaining 21 families corresponded to items found less than 5% of the time. More than half of these 38 items belong to herbs (56.76%, 21 species), followed by trees (21.62%, eight species), lianas (16.22%, six species) and shrubs (5.41%, two species).

Almost three-quarters of items are sold exclusively dry (72.97%), in contrast to all the plants sold (including non-pharmacopoeia ones), where fresh plants were more frequent (almost half) and dry plants represented about one-third of the total. Most frequent plant parts inventoried included fruits (28.95%), roots (26.32%) and seeds (18.42%), followed by far by rhizomes (7.89%), flowers/inflorescences (7.89%) and bulbs (5.26%). Other plant parts represented less than 5% of the items. Of the 38 items, 89.19% of the species have their centres of distribution or domestication in Central and Eastern Asia, and only four, elsewhere showing an even more conserved flora for plants included in the Chinese Pharmacopoeia [12, 32]. Considering that the first volume of the Chinese Pharmacopoeia 2015 [12] comprises over 580 individual plant species (or, in a few cases, extremely similar groups of species) [42], our results indicate that at least 7.3% of these taxa can be found in Fondo’s food stores, and probably more. Relevant nutritional and medicinal properties of the inventoried plants included high contents of vitamins, minerals and fibres, low contents of fats and carbohydrates (except for seeds and reserve organs, respectively) along with a wide spectrum of medicinal properties and treatment of multiple ailments depending on the specific plant product [19].

### Applications and implications: biocultural education and multicultural dialogue

As a derivation of the academic research initiated in 2015, additional publications and outreach activities were developed in collaboration with the *Flavours of the world fair* local partners (Additional file 1). In total, 23 ethnobotanical monographs, 11 ethnoculinary posters, three scientific publications ([43, 44] and the present one), four outreach community events, one bachelor’s degree final project [19], and yearly continuing education courses on gastronomic ethnobotany have derived from the start of this research line in ethnic urban ethnobotany (Table 3, Additional file 3). Overall, these numerous activities had a significant outreach and engagement, with about 1750 people gathered in public events and participating in learning and knowledge sharing activities, along with at least 2500 reads for written materials (monographs, posters and academic papers). These also facilitated the interaction of a diverse and somewhat disparate group of institutions (general public, commercial unions, local governments, universities, public libraries, linguistic institutions, local stores and professional cooks), which probably would have not had this opportunity otherwise.

**Table 3:** Selection of ethnobotanical materials for educational purposes and activities co-organized in and with the local community

Activity/Material	Year/s	Type	Collaborators	Comments [and reference, if applicable]
Bachelor’s degree final project in Pharmacy on the plant diversity of Fondo’s ethnic stores	2014–2015	Final undergraduate degree project	UB, IBB, Fondo’s inventoried stores	The student’s bachelor’s degree project was graded with honours [19]
Flavours of the world fair (4th edition)	2015	One-day outdoor gastronomic event	Flavours of the world fair organizing committee	Participation with one stand to promote and disseminate the academic work carried out in the neighbourhood
Ethnobotanical monographs	2015	Non-academic publication	Flavours of the world fair organizing committee	Ethnobotanical monographs of 10 locally-sold plants distributed during the fair
Fondo’s plants- Ethnobotanical poster	2015	Non-academic publication	UB, IBB	Poster with ethnoculinary information for 13 locally-sold plants
Presentation of ethnobotanical research results in Fondo’s public library	2015	Non-academic colloquium	Flavours of the world fair organizing committee	
Flavours of the world fair (5th edition)	2016	One-day outdoor gastronomic event	Flavours of the world fair organizing committee	Participation with one stand to promote and disseminate the academic work carried out in the neighbourhood
Ethnobotanical monographs	2016	Non-academic publication	Flavours of the world fair organizing committee	Ethnobotanical monographs of 13 locally-sold plants distributed during the fair
Food and culture- Ethnobotanical posters	2016	Non-academic publication	UB, IBB	10 posters on gastronomic ethnobotany
Conference proceeding book chapter on the role that ethnic food plants play in urban transculturation	2017	Academic publication	UB, IBB, Fondo’s inventoried stores	Conference proceeding on the classification of unelaborated products in cooking presented at ICEB 2014 congress in Córdoba, Spain [43]
Article on the classification of unelaborated culinary products	2017	Academic publication	Torribera Food Studies Campus, UB-Bullipèdia partners	Co-authored with academics and chefs [44]
Classification of unelaborated culinary products	2019	Conference presentation	Torribera Food Studies Campus, UB-Bullipèdia partners	Science and cooking
Plants connect us. Ethnobotany, language, cooking and society	2020	Academic colloquium	ODELA, UB, IBB, L’H-CLN, Fondo’s Public Library, Wenzhou restaurant	2 h online event discussing on urban ethnobotany of Santa Coloma de Gramenet
Gaudir UB university extension courses	2015–2022	Teaching in lifelong learning	UB, IBB	University extension courses on gastronomic ethnobotany from around the world including a field trip to Fondo
Article on the research results from 2014–2022	2023	Academic publication	UB, IBB	This current publication

*UB* University of Barcelona, *IBB* Botanical Institute of Barcelona, *ODELA* The Food Observatory, *L’H-CLN* L’Heura-Centre for Linguistic Normalization

At the neighbourhood level, the multiplicity of non-academic activities promoted over the years allowed to encourage the mixing and interaction of cultures, of different ages and genders, and the exchange of knowledge and experience, which consolidates a sense of belonging, respect and recognition of the other, while facilitating meeting and participation spaces around food and plants. Academic activities around ethnobotany, including colloquia and publications, along with non-academic materials and events on plant-human relationships over the period of 2015–2023, including the current publication, have allowed to reach different publics of different ages, origins and backgrounds. For such knowledge valorisation and dissemination, cooperation with various social partners has been essential as it fosters a network of contacts and experience that creates value for society and permits the dissemination of scientific knowledge to a broad audience.

A relevant indirect outcome of our research was the realization that the naming and labelling of the studied plants were obscure, in most cases lacking, at least in Catalan or Spanish. As many of these plants do not have vernacular names in Catalan (or other neighbouring languages such as Spanish or French) many of the labels are written in Chinese characters, contravening local regulations on adequate labelling of products, and, which is relevant, preventing a relevant part of local people to understand the indications. This is the case for example of longan (*Dimocarpus longan*) and lotus (*Nelumbo nucifera*). In addition, certain translations are not fully taxonomically or botanically adequate, showing the importance of including botanists in the formation of neologisms for food plants, their parts or their derivatives. As an example, *Brassica rapa* subsp. *chinensis* (L.) Kitam. is known as ‘bleda xinesa’, in Catalan, which in English would be literally translated into Chinese chard (chard corresponding to *Beta vulgaris* var. *cicla* L., Amaranthaceae) although being phylogenetically more closely related to cabbages. Similarly, in the case of the rhizome of *Nelumbo nucifera* Gaertn., it is considered a root by most vendors and consumers while being a modified stem in botanical terms. In other cases (e.g., *Atractylodes macrocephala* Koidz.), no Catalan name exists, nor from any of its neighbouring Romance languages. These nomenclatural questions point to the importance of adequate plant identification and naming (botanically and vernacularly) as well as appropriate product labelling, and the needed efforts by the governmental administrations towards these goals.

Another significant realization was that little has been researched in the nature and functions of ethnic markets and exotic plants in the metropolitan area of Barcelona, with only a few publications available [43, 45, 46]. Our research points to the importance of ethnic stores for food and medicine provisioning in urban areas, especially for their richness of fresh foods, preserves, and plant foods with various culinary functions (spices, teas, soups, stews, amongst others). Many of the inventoried plants have scientifically renowned nutritional and medicinal properties, indicating that such exotic products may favour a healthy and balanced diet [19, and references therein] and that can play a role in intercultural dialogue and ethnobotany in education, as has been proven elsewhere [33, 47].

Under these conditions, stores become spaces for the exchange of knowledge, ingredients replacement, and the naming of new and unknown species, where processes of hybridization occur simultaneously between and within different ethnobotanical and ethnogastronomic traditions, and where cross-cultural adaptation strategies unravel in different ways [41, 48–51]. In parallel, associated activities surrounding plant-human interactions promoted by local institutions allow for making ethnobotany more visible and known to everyone participating in the programme, in our case, with an outreach of several thousands of people over nine years.

These multiple applications point to the role that ethnobotany can play in co-learning processes and the co-creation of biocultural knowledge between different actors and institutions such as those presented in this manuscript. As has been pointed out in various socioenvironmental arenas, the co-creation of knowledge fosters participatory learning and development [52], can help in co-creating transformative socioecological tools in multicultural settings [53] and facilitate collaborative knowledge generation for public awareness and policy-making [54].

Future ethnobotanical focus should be given to aspects of production, consumption and exchange, as well as on the study of plant-human dynamics with the promotion of dissemination and outreach activities that bring socioenvironmental relations closer to the public. Getting involved in other local programmes and with additional stakeholders, as well as concerning primary and secondary schools would clearly facilitate such focal points.

Additional research could be linked to botanical, ethnobotanical, phytochemical and nutritional analyses of the recently-arrived plants, along with microscopic and molecular studies, when necessary, for improved identification. Moreover, understanding the sustainability of the collection and production of these plants would be needed, as it is unclear how these products, especially those of medicinal use derived from wild plants, have been collected; therefore there should be caution in choosing them, as they may have been harvested in an unsustainable manner rather than having been sustainably collected or cultivated, either organically or not [55, 56].

## Conclusions

The present research shows that multicultural cities—and the interactions between people and plants occurring in them—are highly interesting spaces for ethnobotanical explorations and investigations, as well as to develop biocultural learning programmes and socioenvironmental development, both at the academic and non-academic levels, where people and plant interactions are at the centre. From the implications and applications of introducing a large number of foreign taxa in metropolitan markets and stores to phenomena of hybridization, the need to promote and strengthen biocultural learning in different societal sectors is advocated from these results.

This article also highlights actions promoting access to knowledge through experimentation and innovative and creative methodologies in a collaborative environment open to citizens, along with some of the tensions arising in multicultural settings, with the necessary negotiations between communities for increased integration and exchange.

We expect that over the years and with increased urbanization, ethnobotanical dissemination and outreach become complementary tools for environmental education, social inclusion and multicultural exchange. This would facilitate, amongst others, the bridging between tradition and innovation.

### Supplementary Information

**Additional file 1:** Participants and partners of outreach activities linked to the present paper in Fondo neighbourhood, Santa Coloma de Gramenet, metropolitan area of Barcelona.

**Additional file 2:** Inventory of vascular plants in ethnic stores of Fondo, Santa Coloma de Gramenet. This file includes all data relating to the species collected during the botanical prospections carried out in local stores.

**Additional file 3:** Images of several of the outputs and applied activities developed with other local partners, after the initial plant inventories from a sample of Chinese food stores.

## Data Availability

All data generated or analysed during this study are included in this published article [and its supplementary information files].

## Abbreviations

ChP: Chinese Pharmacopoeia
CRAI-UB: Centre de Recursos per a l’Aprenentatge i la Investigació de la UB (UB’s Learning and Research Resources Centre)
IBB: Institut Botànic de Barcelona (Botanical Institute of Barcelona)
L’H-CLN: L’Heura-Centre de Normalització Lingüística (L’Heura-Centre for Linguistic Normalization)
ODELA: Observatori de l'Alimentació (The Food Observatory)
TCM: Traditional Chinese Medicine
UB: Universitat de Barcelona (University of Barcelona)
