# Supplementary material for: East meets west: using ethnobotany in ethnic urban markets of Barcelona metropolitan area (Catalonia) as a tool for biocultural exchange
Source: J Ethnobiol Ethnomed. 2023 Dec 17;19:63. doi: 10.1186/s13002-023-00636-x (PMC10726630; doi:10.1186/s13002-023-00636-x)
Supplement: Supplementary file 1 — Additional file 1: Participants and partners of outreach activities linked to the present paper in Fondo neighbourhood, Santa Coloma de Gramenet, metropolitan area of Barcelona. [file 13002_2023_636_MOESM1_ESM.docx]

**Additional File 1** Participants and partners of outreach activities linked to the present paper in Fondo neighbourhood, Santa Coloma de Gramenet municipality, metropolitan area of Barcelona

| **Participants** | **Observations** |
| --- | --- |
| Botany laboratory (Faculty of Pharmacy and Food Science, University of Barcelona) and Botanical Institute of Barcelona | Responsible for designing and carrying out ethnobotanical research on ethnic food plants in local neighbourhood stores and producing dissemination materials and activities.  Collaborator in the 4^th^ (2015) and 5^th^ (2016) food fair *Flavours of the world*. |
| Torribera Food Studies Campus, University of Barcelona | Logistic support for ethnobotanical research.  Member of the Cuisines of the world commission through the Food Library (CRAI-UB), the UB-Bullipèdia Unit and the Science and Cooking Studies and Research Unit of the Food Studies Campus, and collaborator in the yearly food fair *Flavours of the world*. |
| The Food Observatory (ODELA), University of Barcelona | Collaboration in an academic colloquium around the project in 2020.  ODELA is an interdisciplinary research team specialized in the management of knowledge about food. |
| Fondo Comerç | Local retailers association, many of which are of Chinese origin.  Member of the Cuisines of the world commission and main organizer of the yearly food fair *Flavours of the world* (2012-2019)*.* Superseded by Agrupació del Comerç i la Indústria de Santa Coloma in 2017 to 2019 editions. |
| L’Heura- Centre for Linguistic Normalization | Catalan language terminological assessment and normalization (including neologisms), responsible for philological support.  Member of the Cuisines of the world commission and collaborator in the yearly food fair *Flavours of the world.* |
| Fondo’s Public Library | Holds extensive bibliography on food and cooking and organizes workshops in its kitchen.  Member of the Cuisines of the world commission and collaborator in the yearly food fair *Flavours of the world*. |
| Santa Coloma de Gramenet City Hall | Local government at the municipality level.  Member of the Cuisines of the world commission, collaborator and main funder in the yearly food fair *Flavours of the world.* |
| Sponsors and private sector | Local small businesses.  Co-founders of the yearly food fair *Flavours of the world*. |
