# Supplementary material for: East meets west: using ethnobotany in ethnic urban markets of Barcelona metropolitan area (Catalonia) as a tool for biocultural exchange
Source: J Ethnobiol Ethnomed. 2023 Dec 17;19:63. doi: 10.1186/s13002-023-00636-x (PMC10726630; doi:10.1186/s13002-023-00636-x)
Supplement: Supplementary file 2 — Additional file 2: Inventory of vascular plants in ethnic stores of Fondo, Santa Coloma de Gramenet. This file includes all data relating to the species collected during the botanical prospections carried out in local stores. [file 13002_2023_636_MOESM2_ESM.docx]

**Additional file 2** Inventory of vascular plants in ethnic stores of Fondo neighbourhood, Santa Coloma de Gramenet municipality. This file includes all data relating to the species collected during the botanical prospections carried out in local stores

| **Family^1^** | **Species^2^** | **Voucher ID** | **Catalan name^3^** | **Spanish name^4^** | **English name^5^** | **Life form^6^** | **Level of preparation** | **Plant part used** | **Centre of diversification^7^** |
| --- | --- | --- | --- | --- | --- | --- | --- | --- | --- |
| Acanthaceae | *Strobilanthes cusia* (Nees) Kuntze | BCN-E 408 | N.E. | N.E. | N.E. | Herb | Dry (mixed with *Isatis tinctoria*) | Aerial parts | N.F. |
| Amaranthaceae | *Spinacia oleracea* L. | BCN-E 427 | Espinacs | Espinaca | Spinach | Herb | Fresh | Leaf | C. Asia |
| Amaryllidaceae | *Allium ampeloprasum* L. var. *porrum* (L.) J.Gay | BCN-E 433 | Porro | Puerro | Leek | Herb | Fresh | Leaf | S. Europe, Asia Minor, Caucasus to Iran and N. Africa |
|  | *Allium cepa* L. | BCN-E 489 | Ceba | Cebolla | Onion | Herb | Fresh | Bulb | C. Asia and esp. NW. India, Afghanistan, Uzbekistan and W. Tien Shan |
|  | *Allium sativum* L. | BCN-E 432 | All | Ajo | Garlic | Herb | Fresh/Dry | Bulb/Aerial parts | N. China |
|  | *Allium schoenoprasum* L. | BCN-E 533 | Cebollí | Cebollino | Chives | Herb | Fresh | Floral stem | Europe, Asia and N. America |
|  | *Allium tuberosum* Rottler ex Spreng. | BCN-E 429 | Cibulet de la Xina | Cebollino ajo | Garlic chives | Herb | Fresh | Stem | E. Mongolia to Japan, the Philippines and through Thailand to N. India |
| Anacardiaceae | *Anacardium occidentale* L. | BCN-E 452 | Anacard | Anacardo | Cashew | Tree | Dry | Seed | Trop. America from Mexico to Peru and Brazil and also the W. Indies. |
|  | *Mangifera indica* L. | BCN-E-542 | Mango | Mango | Mango | Tree | Fresh | Fruit | E. India and Bangladesh |
| Apiaceae | *Angelica sinensis* (Oliv.) Diels | BCN-E 411 | Angèlica de la Xina | Angélica china | Female ginseng | Herb | Dry | Root | N.F. |
|  | *Apium graveolens* L. var. *secalinum* Alef. | BCN-E 531 | Api | Apio | Celery | Herb | Fresh | Leaf | Mediterranean basin |
|  | *Coriandrum sativum* L. | BCN-E 438 | Coriandre | Cilantro, culantro | Coriander | Herb | Fresh | Leaf | Mediterranean basin and W. Asia. |
|  | *Daucus carota* L. subsp. *sativus* (Hoffm.) Arcang. | BCN-E 535 | Pastanaga | Zanahoria | Carrot | Herb | Fresh | Root | Afghanistan |
|  | *Ligusticum striatum* DC. | BCN-E 396 | Api bord de Sichuan | Apio de monte de Szechuan | Szechuan lovage | Herb | Dry | Root | N.F. |
|  | *Petroselinum crispum* (Mill.) Fuss | BCN-E 536 | Julivert | Perejil | Parsley | Herb | Fresh | Aerial parts | S. Europe |
| Aquifoliaceae | *Ilex kudingcha* C.J.Tseng | BCN-E 528 | N.E. | Té Kuding | Kuding | Tree | Dry | Leaf | N.F. |
| Araceae | *Colocasia antiquorum* Schott | BCN-E 414 | Taro | Taro, edo | Eddoe, eddo | Herb | Fresh | Rhizome | SE. Asia |
|  | *Colocasia esculenta* (L.) Schott | BCN-E 413 | Taro | Taro, malanga | Taro, dasheen | Herb | Fresh | Rhizome | SE. Asia |
| Araliaceae | *Panax ginseng* C.A.Mey. | BCN-E 524 | Ginseng | Ginseng | Ginseng | Herb | Dry | Root | N. China, Manchuria, Korea |
| Arecaceae | *Cocos nucifera* L. | BCN-E 482 | Cocoter | Coco | Coconut | Herb* | Fresh | seed | SE. Asia, Indonesia and W. Pacific islands. |
| Asparagaceae | *Rohdea japonica* (Thunb.) Roth | BCN-E 407 | N.E. | N.E. | Sacred lilly | Herb | Dry | Root | N.F. |
| Asteraceae | *Atractylodes macrocephala* Koidz. | BCN-E 404 | N.E. | N.E. | N.E. | Herb | Dry | Rhizome | N.F. |
|  | *Glebionis coronaria* (L.) Cass. ex Spach | BCN-E-544 | Crisantem, sordonaia | Crisantemo | Chrysanthemum greens | Herb | Fresh/Dry | Leaf/Inflorescence | China |
|  | *Glebionis segetum* (L.) Fourr. | BCN-E-543 | Ull de bou | Corona de rey | Corn marigold | Herb | Fresh | Leaf | Europe and Asia |
|  | *Lactuca sativa* L. subsp. *asparagina* (L.H.Bailey) Janch. | BCN-E 434 | Enciam espàrrec | Lechuga espárrago | Celtuce | Herb | Fresh | Aerial parts | N.F. |
|  | *Lactuca sativa* L. subsp. *longifolia* (Lam.) Alef. | BCN-E 538 | Enciam llarg | Lechuga | Lettuce | Herb | Fresh | Aerial parts | Middle East, Mediterranean basin |
| Brassicaceae | *Brassica juncea* (L.) Czern. var. *rugosa* (Roxb.) Kitam. | BCN-E 529 | Mostassa xinesa | Mostaza china | Chinese mustard | Herb | Fresh/Preserved | Aerial parts | Africa or C. Asia-Himalayas with secondary centres of diversity in India, China and Caucasia |
|  | *Brassica juncea* (L.) Czern. var. *tsatsai* Z.I.Mao | BCN-E 476 | Mostassa xinesa | Mostaza china | Chinese mustard | Herb | Preserved | Aerial parts | Africa or C. Asia-Himalayas with secondary centres of diversity in India, China and Caucasia |
|  | *Brassica oleracea* L. f. *pyramidalis* (Mill.) O.E.Schulz | BCN-E 473 | Col | Col picuda | Spring cabbage | Herb | Fresh | Aerial parts | Asia minor |
|  | *Brassica oleracea* L. var. *alboglabra* L. | BCN-E 472 | Bròquil xinès | Brécol chino, col verde china | Chinese broccoli | Herb | Fresh | Aerial parts | China |
|  | *Brassica oleracea* L. var. *botrytis* L. | BCN-E 468 | Coliflor | Coliflor | Cauliflower | Herb | Fresh | Aerial parts | Mediterranean basin and Atlantic fringe of Europe |
|  | *Brassica oleracea* L. var. *gongylodes* L. | BCN-E-546 | Colrave | Colirrábano | Kohlrabi | Herb | Fresh | Aerial parts | N.F. (probably Mediterranean basin and Atlantic fringe of Europe) |
|  | *Brassica rapa* L. subsp. *nipposinica* (L.H.Bailey) Hanelt | BCN-E 436 | N.E. | Mostaza de hoja, mostaza japonesa | Japanese mustard greens | Herb | Fresh | Aerial parts | China |
|  | *Brassica rapa* L. subsp. *pekinensis* (Lour.) Hanelt | BCN-E 471 | Col xinesa | Col china | Chinese cabbage | Herb | Fresh | Aerial parts | China |
|  | *Brassica rapa* L. var. *chinensis* (L.) Kitam. | BCN-E 435 | Bleda xinesa | Bok choi, pak choi | Bok choi, pak choi | Herb | Fresh | Aerial parts | China |
|  | *Brassica rapa* L. var. *parachinensis* (Bailey) Hanelt | BCN-E 530 | N.E. | N.E. | Chinese flowering cabbage | Herb | Fresh | Aerial parts | China |
|  | *Isatis tinctoria* L. | BCN-E 523 | Glast, pastell | Hierba pastel, glasto | Woad | Herb | Dry (mixed with *Strobilanthes cusia*) | Root | N.F. |
|  | *Nasturtium officinale* R.Br. | BCN-E 428 | Morritort, creixen | Berro de agua | Watercress | Herb | Fresh | Leaf | W. Asia, S. Europe and Britain |
|  | *Raphanus sativus* L. | BCN-E 534 | Rave | Rábano | Radish | Herb | Fresh | Root | E. Mediterranean basin |
|  | *Raphanus sativus* L. var. *longipinnatus* L.H. Bailey | BCN-E 420 | Rave daikon | Rábano daikon | Daikon radish | Herb | Fresh | Root | China and Japan |
| Cactaceae | *Hylocereus undatus* (Haw.) Britton & Rose | BCN-E 458 | Flor de calze | Pitahaya | Pitaya | Herb* | Fresh | Fruit | Mexico |
| Campanulaceae | *Codonopsis pilosula* (Franch.) Nannf. | BCN-E 412 | N.E. | N.E. | N.E. | Liana | Dry | Root | China |
| Caprifoliaceae | *Lonicera japonica* Thunb. | BCN-E 469 | Xuclamel o lligabosc japonès | Madreselva japonés | Japanese honeysuckle | Liana | Dry | Flower | N.F. |
| Chenopodiaceae | *Beta vulgaris* L. var. *cicla* L. | BCN-E 539 | Bleda | Acelga | Chard | Herb | Fresh | Leaf | It very likely derives from early introductions from the Near East via Afghanistan, whereas the table beetroot was introduced later from Europe |
| Convolvulaceae | *Ipomoea batatas* (L.) Lam. 'Okinawa' | BCN-E 479 | Moniato | Boniato | Sweet potato | Liana | Fresh | Root | Unkn. (probably C. America) |
| Cucurbitaceae | *Benincasa hispida* (Thunb.) Cogn. var. *chieh-gua* F.C.How | BCN-E 405 | Carbassa pelosa | Calabaza de la cera, calabaza blanca, calabaza china | Wax gourd | Liana | Fresh | Fruit | Tropical Asia |
|  | *Cucumis sativus* L. | BCN-E 487 | Cogombre | Pepino | Cucumber | Liana | Fresh | Fruit/ Seed | India |
|  | *Cucurbita moschata* Duchesne | BCN-E 477 | Carabassera moscada | Calabaza cacahuete | Butternut squash | Liana | Fresh | Fruit | From Mexico to Peru |
|  | *Lagenaria siceraria* (Molina) Standl. | BCN-E 475 | Carabassera vinatera | Calabaza del peregrino, calabaza vinatera | Calabash, bottle gourd | Liana | Fresh | Fruit | Unkn. (probably Papua New Guinea) |
|  | *Luffa acutangula* (L.) Roxb. | BCN-E 422 | Esponges | Esponja | Angled luffa, sponge gourd | Liana | Fresh | Fruit | India |
|  | *Momordica charantia* L. | BCN-E 406 | Cogombre tropical | Pepino amargo | Bitter melon, bitter gourd | Liana | Fresh | Fruit | Old world tropics |
|  | *Sechium edule* (Jacq.) Sw. | BCN-E 486 | Sequi | Chayote | Chayote | Liana | Fresh | Fruit | C. America |
|  | *Siraitia grosvenorii* (Swingle) C.Jeffrey ex A.M.Lu & ZhiY.Zhang | BCN-E 391 | N.E. | N.E. | Monk fruit | Liana | Dry | Fruit | N.F. |
| Cyperaceae | *Eleocharis dulcis* (Burm.f.) Trin. ex Hensch. | BCN-E 448 | Castanya d'aigua | Castaña de agua | Water chestnut | Herb | Fresh | Rhizome | S. China (W. Africa to India, China, Japan, New Caledonia) |
| Dioscoreaceae | *Dioscorea cayennensis* Lam. subsp. *rotundata* (Poir.) J. Miège | BCN-E 456 | Nyam africà | Ñame africano | African yam | Liana | Fresh | Tuber | W. Africa |
|  | *Dioscorea oppositifolia* L. | BCN-E 421 | Nyam japonès | Ñame japonés, ñame chino | Asian yam | Liana | Fresh | Tuber | China |
| Euphorbiaceae | *Manihot esculenta* Crantz | BCN-E 483 | Mandioca | Yuca, mandioca | Cassava | Herb | Fresh | Root | C. and S. America |
| Fabaceae | *Arachis hypogaea* L. | BCN-E 449 | Cacauet | Cacahuete | Peanut | Herb | Dry | Seed | S. America |
|  | *Astragalus propinquus* Schischkin | BCN-E 416 | Astràgal de Mongòlia | Astrágalo de Mongolia | Mongolian milkvetch | Herb | Dry | Root | N.F. |
|  | *Glycine max* (L.) Merr. | BCN-E 463 | Soia | Soja | Soy | Herb | Dry/Coagulated/Fresh | Seed/Seedling | China |
|  | *Glycyrrhiza uralensis* Fisch. | BCN-E 403 | Regalèssia | Regaliz | Chinese liquorice | Herb | Dry | Root | N.F. |
|  | *Lablab purpureus* (L.) Sweet | BCN-E 459 | Mongeta egípcia | Zarandaja, judía de Egipto | Hyacinth bean | Herb | Fresh | Fruit | Tropical Africa |
|  | *Phaseolus vulgaris* L. | BCN-E 532 | Mongetera | Judía | Bean | Liana | Dry | Fruit/ Seed | C. and S. America |
|  | *Pisum sativum* L. | BCN-E 453 | Pèsol, tirabec | Guisante, tirabeque | Pea | Liana | Fresh | Fruit | Middle East, Mediterranean basin |
|  | *Vicia faba* L. | BCN-E 419 | Fava | Haba | Fava bean | Herb | Dry | Seed | SW. Asia or Mediterranean basin |
|  | *Vigna angularis* (Willd.) Ohwi & H.Ohashi | BCN-E 464 | Mongeta azuki | Judía adzuki | Adzuki bean | Liana | Dry | Seed | C. China |
|  | *Vigna mungo* (L.) Hepper | BCN-E 466 | Mongeta mungo (negra) | Judía mungo (negra) | Mungo bean | Herb | Dry | Seed | India |
|  | *Vigna radiata* (L.) R.Wilczek | BCN-E 465 | Mongeta mung (verda) | Judía mung (verde), soja verde | Mung bean, green gram | Herb | Dry | Seed | India and Burma |
|  | *Vigna unguiculata* (L.) Walp. subsp. *sesquipedalis* (L.) Verdc. | BCN-E 451 | Caragirat | Judía de metro | Asparagus bean | Liana | Fresh | Fruit | W. and C. Africa |
| Fagaceae | *Castanea mollissima* Blume | BCN-E 522 | Castanyer de la Xina | Castaño chino | Chinese chestnut | Tree | Dry | Seed | N. and W. China |
| Illiciaceae | *Illicium verum* Hook.f. | BCN-E 439 | Badiana | Anís estrellado | Star anise | Tree | Dry | Fruit | SE. Asia |
| Juglandaceae | *Juglans regia* L. | BCN-E 445 | Nou | Nuez | Walnut | Tree | Dry | Seed | C. Asia |
| Lamiaceae | *Prunella vulgaris* L. | BCN-E 418 | Prunel·la vulgar | Consuelda menor | Common self-heal | Herb | Dry | Inflorescence | N.F. |
|  | *Salvia miltiorrhiza* Bunge | BCN-E 526 | Sàlvia vermella | Salvia roja | Red sage | Herb | Dry | Root | N.F. |
| Lauraceae | *Cinnamomum cassia* (L.) J.Presl | BCN-E 398 | Càssia, cínamom xinès | Canela de la China, canela de Manila | Chines cassia | Tree | Dry | Bark | S. China |
| Lilliaceae | *Lilium brownii* F.E.Br. ex Miellez | BCN-E 461 | Lliri | Lirio | Brown's lily | Herb | Dry | Bulb | N.F. |
| Lythraceae | *Trapa natans* L. | BCN-E 454 | N.E. | N.E. | Water caltrop | Herb | Dry | Seed | Europe to Asia |
| Malvaceae | *Abelmoschus esculentus* (L.) Moench | BCN-E 393 | Ocra | Okra, quimbombó | Okra | Shrub | Fresh | Fruit | Tropical Asia |
|  | *Scaphium affine* (Mast.) Pierre | BCN-E 480 | N.E. | N.E. | Malva nut tree | Herb | Dry | Fruit | N.F. |
| Musaceae | *Musa* ×*paradisiaca* L. AAB group | BCN-E 484 | Bananer groc | Plátano macho | Plantain | Herb | Fresh | Fruit | Malay peninsula |
|  | *Musa acuminata* L. AAA group | BCN-E 457 | Bananer | Plátano, banano | Banana | Herb | Fresh | Fruit | Malay peninsula |
| Myricaceae | *Myrica rubra* (Lour.) Siebold & Zucc. | BCN-E 409 | N.E. | Fresa china, arrayán chino | Chinese bayberry | Shrub | Preserved | Fruit | China |
| Nelumbonaceae | *Nelumbo nucifera* Gaertn. | BCN-E 410 | Nelumbe | Loto sagrado | Sacred lotus | Herb | Fresh/Dry | Rhizome/Seed | N. Iran, Transcaucasia and Volga Region |
| Nymphaeaceae | *Euryale ferox* Salisb. | BCN-E 525 | N.E. | Planta gorgona | Prickly waterlily | Herb | Dry | Seed | Tropical Asia |
| Paeoniaceae | *Paeonia lactiflora* Pall*.* | BCN-E 397 | Peònia de jardí, peònia de la Xina | Peonia china | Chinese peony | Herb | Dry | Root | N.F. |
| Passifloraceae | *Passiflora ligularis* Juss. | BCN-E 474 | Granadilla | Granadilla | Sweet granadilla | Liana | Fresh | Fruit | Tropical America |
| Pedaliaceae | *Sesamum idicum* L. | BCN-E 481 | Sèsam | Sésamo | Sesame | Herb | Dry | Seed | Africa |
| Poaceae | *Bambusa vulgaris* Schard. | BCN-E 478 | Bambú | Bambú común | Common bamboo | Herb | Preserved | Young stem | Probably Malaysia or India |
|  | *Coix lachryma-jobi* L. | BCN-E 485 | Llàgrima de Job | Lágrima de San Pedro | Job's tears | Herb | Dry | Seed | S. Asia |
|  | *Cymbopogon citratus* (DC.) Stapf | BCN-E 447 | Herba llimona | Hierba limón | Lemongrass | Herb | Fresh | Aerial parts | Malaysia or Sri Lanka |
|  | Hordeum vulgare L. | BCN-E 467 | Ordi | Cebada | Barley | Herb | Dry | Seed | E. Europe to W. Asia |
|  | *Indocalamus tessellatus*  (Munro) Kengf. | BCN-E 431 | Bambú de fulla gran | Bambú de hoja grande | Large-leaved bamboo | Herb | Dry (envelope for cooking or *zongzi*) | Leaf | N.F. |
|  | *Oryza sativa* L. | BCN-E 424 | Arròs | Arroz | Rice | Herb | Many forms | Seed | S. Asia |
|  | *Saccharum officinarum* L. | BCN-E 541 | Canya de sucre | Caña de azúcar | Sugar cane | Herb | Dry | Stem | New Guinea |
|  | *Zea mays* L. | BCN-E-426 | Blat de moro | Maíz | Corn | Herb | Dry | Fruit | C. America |
| Rhamnaceae | *Ziziphus jujuba* Mill. | BCN-E 415 | Ginjoler | Jujube, azufaifo | Jujube | Tree | Dry | Fruit | C. and S. China |
| Rosaceae | *Crataegus pinnatifida* Bunge | BCN-E 462 | Arç xinès | Espino chino | Mountain hawthorn | Tree | Dry | Fruit | N. China, Korea and Siberia |
|  | *Prunus persica* (L.) Batsch | BCN-E 420 | Préssec | Melocotón | Peach | Tree | Fresh/Dry | Fruit | Tibet and SW. China |
|  | *Pyrus pyrifolia* (Burm.f.) Nakai | BCN-E 425 | Nashi | Pera nashi | Nashi pear | Tree | Fresh | Fruit | Highlands N. and C. China |
|  | *Rosa rugosa* Thunb. | BCN-E 527 | Rosa japonesa | Rosa japonesa | Rugosa rose | Shrub | Dry | Flower | N.F. |
| Rutaceae | *Citrus aurantiifolia* (Christm.) Swingle | BCN-E 537 | Llima | Lima | Lime | Tree | Fresh | Fruit | Malaysian Archipelago or N. India |
|  | *Citrus japonica* Thunb. | BCN-E 392 | Cumquat | Kumquat | Kumquat | Tree | Fresh | Fruit | Japan |
| Sapindaceae | *Dimocarpus longan* Lour. | BCN-E 400 | Longan | Longán | Longan | Tree | Dry | Fruit | N.F. |
|  | *Litchi chinensis* Sonn. | BCN-E 460 | Litxi | Lichi | Lychee | Tree | Dry/ Preserved | Fruit | S. China |
| Scrophulariaceae | *Rehmannia glutinosa* (Gaertn.) DC. | BCN-E 395 | N.E. | N.E. | N.E. | Herb | Dry | Root | N.F. |
| Solanaceae | *Capsicum annuum* L. | BCN-E 446 | Pebrot picant | Pimiento | Pepper | Shrub | Fresh | Fruit | Mexico |
|  | *Capsicum annuum* L. | BCN-E 540 | Pebrot verd | Pimiento verde | Green pepper | Shrub | Fresh | Fruit | Mexico |
|  | *Capsicum frutescens* L. | BCN-E 450 | Xile picant | Chile picante | Chili | Shrub | Fresh/Dry | Fruit | W. Indies and lowland S. America, from S. Bolivia to S. Brazil |
|  | *Lycium chinense* Mill. | BCN-E 470 | Goji | Goji | Goji berry | Shrub | Dry | Fruit | N.F. |
|  | *Solanum melongena* L. | BCN-E 399 | Alberginiera | Berenjena | Eggplant, aubergine | Herb | Fresh | Fruit | India |
| Theaceae | *Camellia sinensis* (L.) Kuntze | BCN-E 440 | Te | Té | Tea | Shrub | Dry | Leaf | Mountains of China, N. of NE. India |
| Zingiberaceae | *Maranta arundinacea* L. | BCN-E 423 | Maranta | Maranta, sagú | Arrowroot | Herb | Fresh/Dry | Rhizome | Northern S. America |
|  | *Zingiber officinale* Roscoe | BCN-E 488 | Gingebre | Jengibre | Ginger | Herb | Fresh | Rhizome | India |

^1 [^[28]; ^2^ [20-23, 25]; ^3^ [24]; ^4^ [26]; ^5^ [26-27]; ^6^ Herbs include succulents, palms and others; ^7^ [30-31]. Abbreviations: N.E.: Non-existent name / N.F.: Not found / Unkn.: Unknown
