# Supplementary material for: East meets west: using ethnobotany in ethnic urban markets of Barcelona metropolitan area (Catalonia) as a tool for biocultural exchange
Source: J Ethnobiol Ethnomed. 2023 Dec 17;19:63. doi: 10.1186/s13002-023-00636-x (PMC10726630; doi:10.1186/s13002-023-00636-x)

**Additional file 3** Images of several of the outputs and applied activities developed with other local partners, after the initial plant inventories from a sample of Chinese food stores.

**a)** Poster (in Catalan) with a selection of Fondo’s Chinese food plants. Includes scientific and common names in Catalan, Spanish and Chinese, along with information on vital form, plant parts used, mode of preparation and nutritional properties.


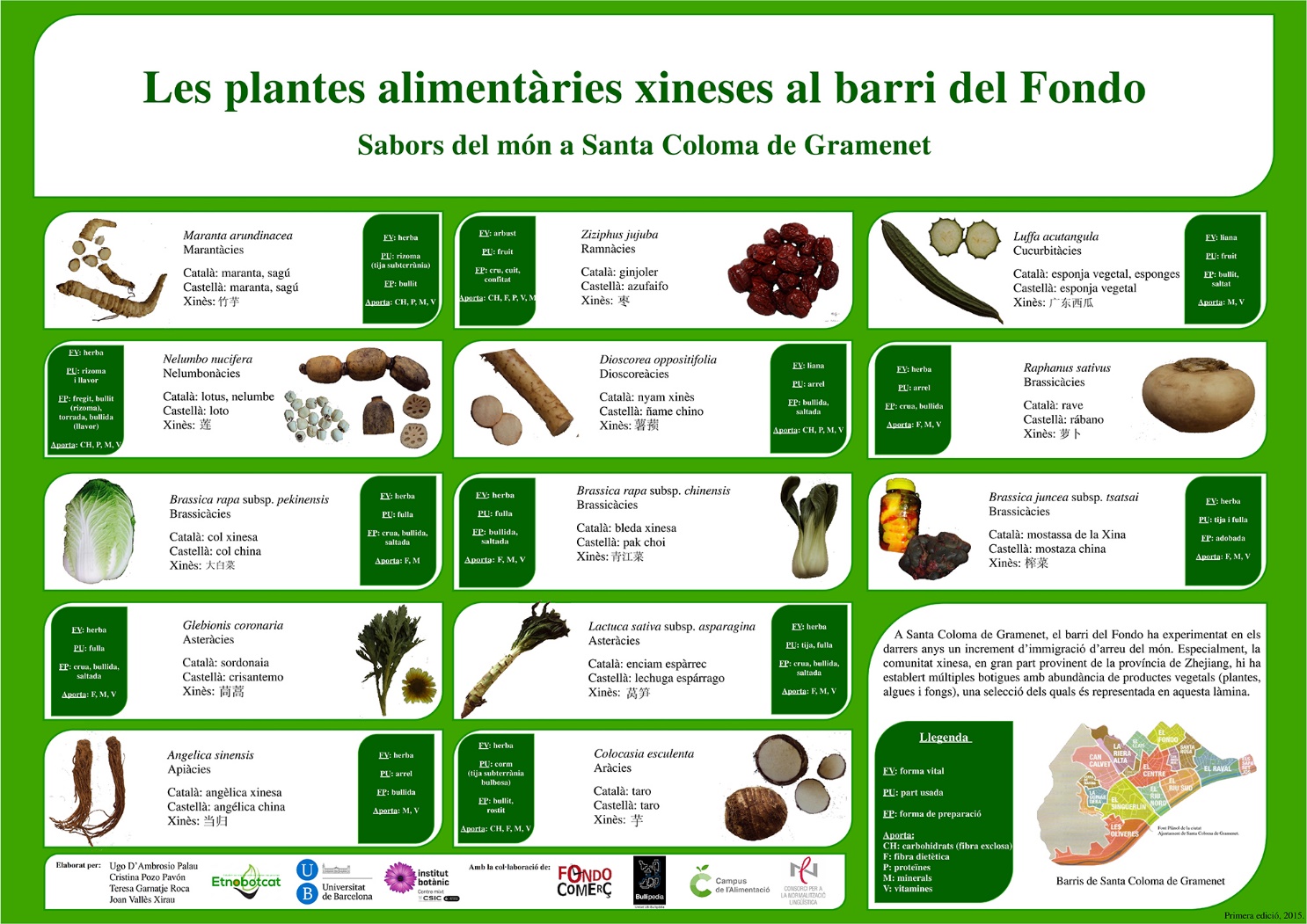


**b)** Example of a monograph (in Catalan) developed by the research team for the *Flavours of the world* fair, 2015. Twenty-three monographs were prepared in total during two consecutive years.


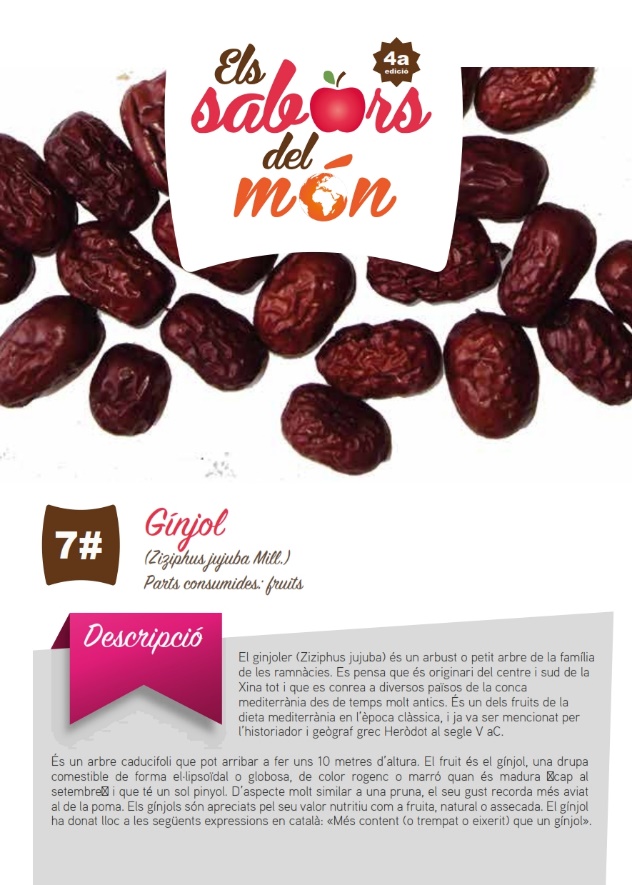

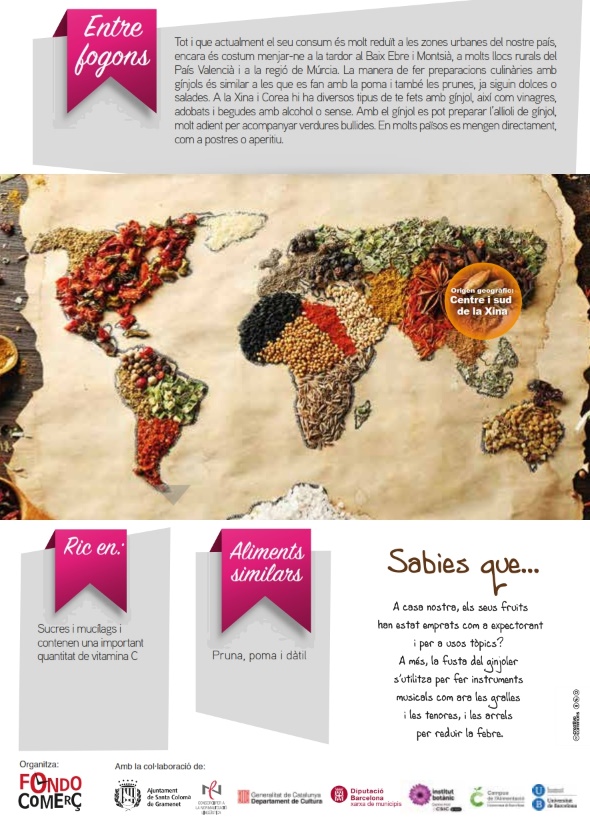


**c)** Poster (in Catalan) with an overview pulses domesticated in Southeast Asia, sub-Saharan African and America. Nine other posters were prepared and shared.


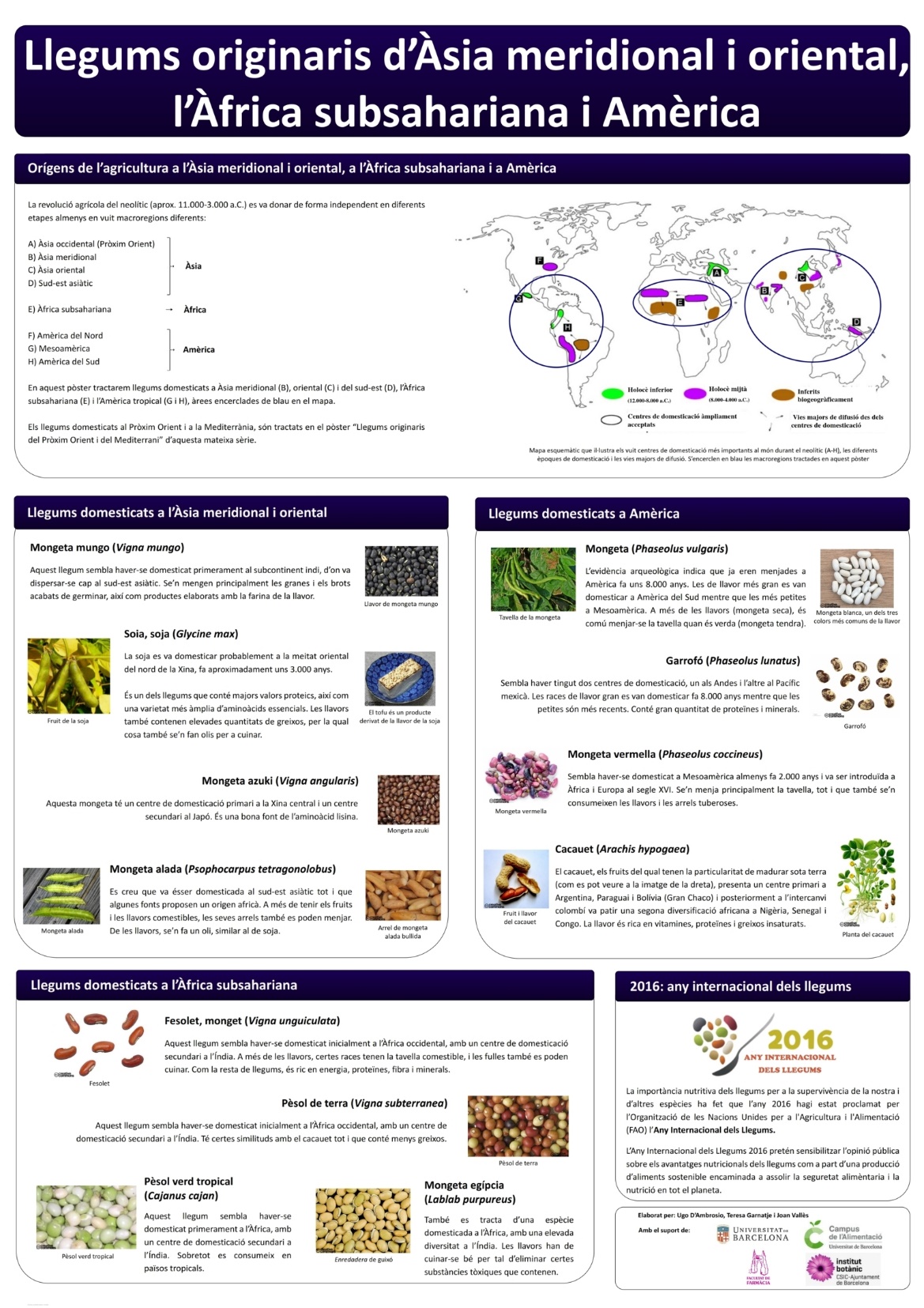


**d)** Presentation of ethnobotanical research results in Fondo’s public library (May 2015) under the local *Cuisines of the world* initiative, in combination with culinary demonstrations by gastronomy students. The presence of cook students and teacher, the mayor of Santa Coloma de Gramenet, one highly-considered local cook, and two co-authors of this paper, shows the twofold approach of the present research, academic and social.


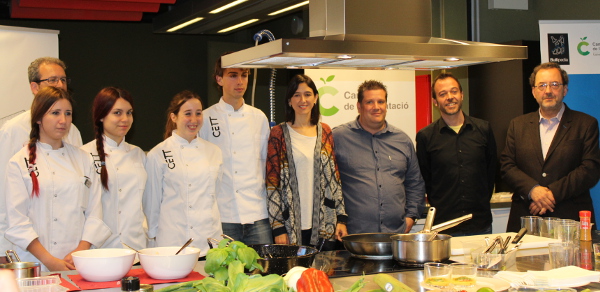


**e)** Images of the 4^th^ edition of the *Flavours of the world* fair, including symposia, tastings and culinary demonstrations (June 2015).


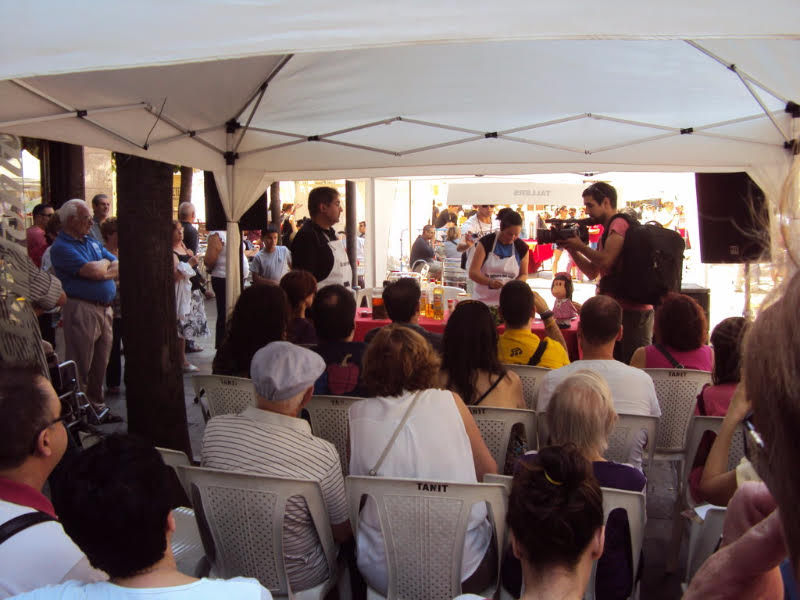

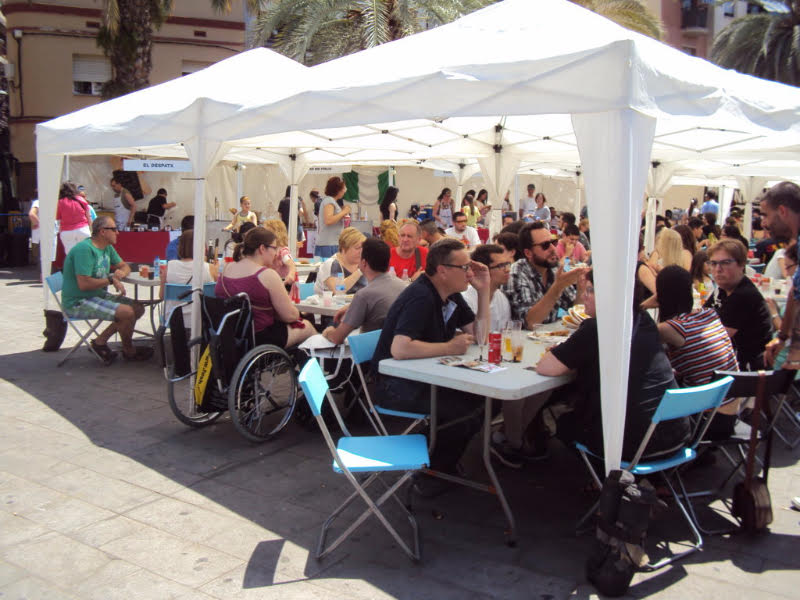

Supplement: Supplementary file 3 — Additional file 3: Images of several of the outputs and applied activities developed with other local partners, after the initial plant inventories from a sample of Chinese food stores. [file 13002_2023_636_MOESM3_ESM.docx]
